# Preliminary Study on Tracing the Origin and Exploring the Relations between Growing Conditions and Isotopic and Elemental Fingerprints of Organic and Conventional Cavendish Bananas (*Musa* spp.)

**DOI:** 10.3390/foods10051021

**Published:** 2021-05-08

**Authors:** Zhijun Wang, Sara W. Erasmus, Saskia M. van Ruth

**Affiliations:** 1 Food Quality & Design Group, Wageningen University & Research, P.O. Box 17, 6700 AA Wageningen, The Netherlands; zhijun.wang@wur.nl (Z.W.); sara.erasmus@wur.nl (S.W.E.); 2 Wageningen Food Safety Research, Wageningen University & Research, P.O. Box 230, 6700 AE Wageningen, The Netherlands

**Keywords:** banana, elements, growing conditions, stable isotopes

## Abstract

The stable isotopic ratios and elemental compositions of 120 banana samples, *Musa* spp. (AAA Group, Cavendish Subgroup) cultivar Williams, collected from six countries (Colombia, Costa Rica, Dominica Republic, Ecuador, Panama, Peru), were determined by isotope ratio mass spectrometry and inductively coupled plasma mass spectrometry. Growing conditions like altitude, temperature, rainfall and production system (organic or conventional cultivation) were obtained from the sampling farms. Principal component analysis (PCA) revealed separation of the farms based on geographical origin and production system. The results showed a significant difference in the stable isotopic ratios (δ^13^C, δ^15^N, δ^18^O) and elemental compositions (Al, Ba, Cu, Fe, Mn, Mo, Ni, Rb) of the pulp and peel samples. Furthermore, δ^15^N was found to be a good marker for organically produced bananas. A correlation analysis was conducted to show the linkage of growing conditions and compositional attributes. The δ^13^C of pulp and peel were mainly negatively correlated with the rainfall, while δ^18^O was moderately positively (R values ~0.5) correlated with altitude and temperature. A moderate correlation was also found between temperature and elements such as Ba, Fe, Mn, Ni and Sr in the pulp and peel samples. The PCA results and correlation analysis suggested that the differences of banana compositions were combined effects of geographical factors and production systems. Ultimately, the findings contribute towards understanding the compositional differences of bananas due to different growing conditions and production systems linked to a defined origin; thereby offering a tool to support the traceability of commercial fruits.

## 1. Introduction

Banana (*Musa* spp.) is one of the most representative fruits which are well-liked by consumers for their flavor and nutritional value. Generally, the cooking banana, also called plantain, is used as a staple food for millions of people in many countries, especially in developing economies, because it is an affordable source of energy [1]. Another kind of popular banana is called the dessert banana, which is used as an everyday fruit because of its soft texture, sweet taste and bioactive components [2]. The dessert banana is typically used as a food companion with products such as breakfast cereals, ice cream, and other desserts apart from just its raw consumption. In the current international market, the Cavendish variety is the most commercialized bananas and consumed world-wide [3]. Cavendish bananas are rich in carbohydrates, vitamins, minerals and dietary fiber, indicating that they have anti-oxidative and anti-ageing activities [4].

In 2019 alone, around 21 million tons of bananas, excluding plantains, were exported from Ecuador, Colombia, Costa Rica, etc. Latin America and the Caribbean are the largest exporting regions, and the European Union (EU), United States and China are the largest global importers [5]. As the quality of the banana is highly dependent on climate and cultivation system, the label of origins is considered an important parameter for quality and price [6]. The production of organic bananas requires stricter management, such as forbidding the use of pesticides, chemical fertilizers, and pesticides [7]. In the case of consumer preferences, the geographical origin and production system play a role in the purchase intent of consumers [8]. However, there are only a few papers about the authentication of the geographical origin and production system of bananas.

In recent years, several papers have been published concerning the geographical origin and organic production of agricultural products [9,10,11]. This is because food fraud and public health incidents occur frequently around the world. Food fraud, mainly known as economically motivated adulteration, is an ongoing problem [12]. Although banana is not an extremely vulnerable target, authenticity research of banana still has important value [13]. The banana is a staple food in many developing countries to ensure household food security. Meanwhile, the income from the banana industry is also important for approximately 400 million workers globally [5]. In recent years, the production of bananas is facing many challenges, such as infectious diseases *Fusarium wilt*, mislabeled organic bananas, sustainable production with less pesticides, etc. A reliable tracing system is helpful in banana chains to protect smallholder farmers, the retailer from unfair competition, and make sure the consumers get what they pay for [14]. Previous studies have reported that the combined application of stable isotopes and elemental compositions could effectively identify the geographical origin of a variety of agricultural products such as grape wine [15], pork [16], honey [17], tea [18], etc. Nevertheless, the measurement of stable isotopes could also reveal if the foods were produced in organic cultivations. The combination of δ^13^C, δ^15^N and δ^18^O could provide a sound solution for rice origin using chemometrics [19]. For bell peppers, the linking of δ^18^O in the sample and local water could indicate the geographical origin [20]. Previous research also confirmed that elemental analysis of Mn, Cr, Sr, Ag and Co could differentiate wines from different Romanian vineyards [21]. In terms of bananas, there are limited papers about the geographical origin of bananas. One relevant study explored the impact of farming type, variety and geographical origin on bacterial community of banana, and was aimed at discriminating organic bananas from conventional ones [22]. Most of the published research focused on the discrimination of geographical origin and production system, while only a few research studies aimed to explore the underlining reasons that foods have various composition differences according to different origins and production systems. There are already studies which reported that the lithological properties of origin could change the element contents of garlic [23], while latitudes had significant influences on the depleted δ^2^H and δ^18^O values of poultry [24]. Our previous study revealed that the isotopic ratios and elemental profiles of bananas were highly related to climate, topography and soil conditions in a single country [25]. To the best of our knowledge, only limited works have studied the relation of growing conditions and the composition of bananas in different countries. In fact, most of the related studies focuses on ways to alter the growing conditions to increase the yield of bananas.

In this preliminary study, the main stable isotope ratios and elemental compositions of banana samples from different origins were characterized with the aim to establish correlations among these fingerprints and the growing conditions of bananas. Banana samples, including pulp and peel, were collected from ten farms located in six different countries in South America. Besides the related growing conditions such as altitude, rainfall and temperatures, some farms cultivated bananas according to an organic and some to a conventional production practices. The stable isotopic ratios and elements of pulp and peel from different countries were firstly characterized through isotope ratio mass spectrometry (IR-MS) and inductively coupled plasma mass spectrometry (ICP-MS). Furthermore, the network between stable isotopic and elemental fingerprints of organic/conventional bananas and their growing conditions were correlated. Due to the limitations in exploring the effects of external (environmental) factors such as harvest year and season together with the use of limited samples numbers, the paper was aimed to provide the feasibility study for the relations between growing conditions and isotopic and elemental fingerprints of bananas.

## 2. Materials and Methods

### 2.1. Sample Collection and Growing Conditions

**Figure 1:**
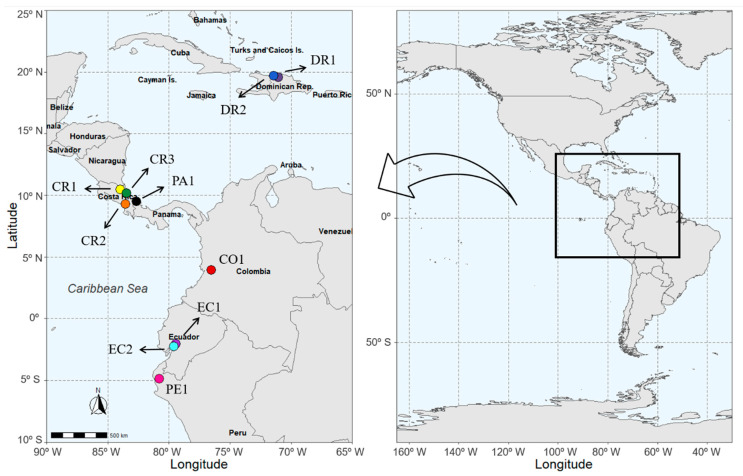
The sampling sites of the collected bananas. CO: Colombia; CR: Costa Rica; DR: Dominican Republic; EC: Ecuador; PA: Panama; PE: Peru; the numbers refer to the individual farms in a specific country.

Banana (*Musa* spp.) samples (AAA Group, Cavendish Subgroup) cultivar (cv.) Williams were collected in a 3-month period from February to April 2018 from ten farms located in six different countries namely, Colombia, Costa Rica, Dominica Republic, Ecuador, Panama, Peru. The locations of the farms, based on their Global Positioning System (GPS) coordinates, are shown in Figure 1. For each farm, twelve bunches from the top position of the banana trees were randomly selected. The bunches were then placed in clean polyethene bags, labelled with the sampling site location and transported in low temperature and dark conditions to Wageningen University & Research. Upon receival of the bunches, two banana fingers were randomly selected from each bunch and combined as one research sample. The sampled bananas were then separated into pulp and peel, cut into slices, freeze-dried and crushed to fine powder. Ultimately, 120 powdered banana pulp and peel samples were collected and stored at −20 °C until IR-MS and ICP-MS analyses. The growing conditions that include the geographical factors and production system data were also obtained from local farms. The overview of the collected banana samples, altitude, monthly mean temperature, annual rainfall and production system data is shown in Table 1.

### 2.2. Determining the Stable Isotope Ratios

The three kinds of stable isotopes (Carbon, Nitrogen and Oxygen) of bananas were performed according to the protocol by Erasmus et al. with slight changes [26]. The banana pulp and peel samples were analyzed for δ^13^C and δ^15^N using an isotope ratio mass spectrometer (Flash 2000 in combination with Delta V advantage, Thermo Scientific, Waltham MA USA). The results were expressed as stable isotope ratios (^13^C/^12^C or ^15^N/^14^N) against Vienna Pee Dee Belimnite (VPDB) for δ^13^C and atmospheric AIR for δ^15^N. The oxygen isotope ratios were performed by a SerCon high-temperature elemental analyzer interfaced with a SerCon 20-20 IR-MS with SysCon electronics (Sercon, Cheshire, UK). The final delta unit (δ^18^O) was expressed as ^18^O/^16^O relative to international standards VSMOW2 (Vienna Standard Mean Ocean Water). For δ^13^C and δ^15^N, the samples were weighed into tin capsule, while 4 × 6 mm silver cups were used for δ^18^O. To reduce the uncertainty and ensure the quality of the measurement, the international atomic energy agency (IAEA) standard samples (IAEA-600 for Carbon, Nitrogen and IAEA-601 for Oxygen) were used. Every 12 samples were interspersed with a standard sample for calibration. All analyses were conducted in triplicate.

### 2.3. Determining the Elemental Compositions

The 11 kinds of elemental compositions (Al, Ba, Cr, Cu, Fe, Mn, Mo, Ni, Rb, Sr and Zn) of banana pulp and peel were determined by ICP-MS [27]. The freeze-dried and fine homogeneous samples (pulp/peel) were digested using a MARS 6 microwave (CEM Corporation, Matthews NC, USA). About 0.8 g sample was accurately weighed into Teflon vessels. For digestion, 3 mL 70% Nitric acid (HNO_3_) and 1 mL 30% Hydrogen peroxide (H_2_O_2_) were added to each vessel. Besides, another vessel only within HNO_3_ and H_2_O_2_ was selected as a blank group. After digestion, the samples were transferred to tubes and filled up to 10 mL with Milli-Q water. Standard solutions were prepared from 1000 mg/L ICP-MS stock solutions (ICP-MS calibration standard, ULTRA Scientific, North Kingstown, Rhode Island). The multi-elemental analyses were performed using a NexION 300D ICP-MS (Perkin Elmer, Waltham, MA, USA). The analytical performance was verified by processing certified reference materials Lichen 482 (Sigma-Aldrich, Buchs, Switzerland), tuning solution A and B (Merck, Darmstadt, Germany). The concentrations of elemental composition in samples were determined using external calibration curves and the rhodium was used as an internal standard.

### 2.4. Statistical Analysis

The compositional data were statistically analyzed using a one-way analysis of variance (ANOVA) and Tukey’s test (multiple-range) to assess the impact of the geographical factors and production system of bananas, as a single categorical factor, on the different stable isotopic ratios and elemental compositions. Means with *p* values below 0.05 were significantly different [28]. The multivariate characterization of the samples concerning stable isotopic ratios and elements, and growing conditions were performed by multiple variable regression and principal component analysis (PCA) [29]. The stable isotopic ratios and elemental compositions were pre-processed using a log transformation before PCA analysis was performed. Pearson correlation coefficients (R) were calculated to evaluate the correlation between growing conditions and banana compositions [30]. Data analyses and the visualizations were conducted using R 4.0.2 (R Foundation for Statistical Computing, Vienna, Austria).

## 3. Results and Discussion

### 3.1. Summary of the Growing Conditions of Banana Farms

**Figure 2:**
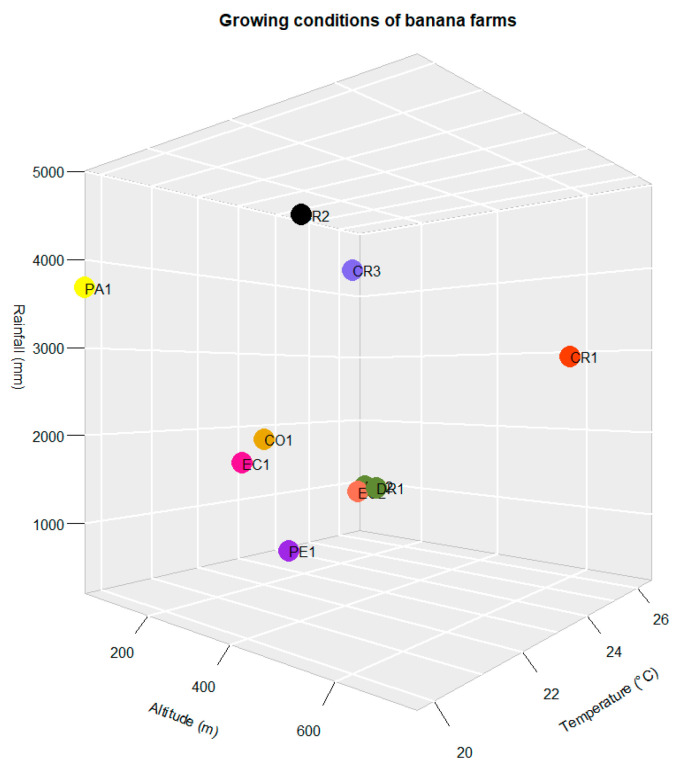
Differences in geographical factors of banana farms from Colombia (CO), Costa Rica (CR), Dominican Republic (DR), Ecuador (EC), Panama (PA), and Peru (PE). The numbers refer to individual farms in a country (please refer to the web version of this article for the interpretation of the colors used in the figure).

The geographical factors (altitude, monthly mean temperature and annual rainfall) and production system (organic and conventional cultivation) were regarded as growing conditions of the banana farms. In total, ten farms from six different countries were selected as sampling sites due to their differences in growing conditions between the different farms (Figure 1). Table 1 shows the six conventional farms (CO1, CR1, CR2, CR3, EC2, PA1) and four organic farms (DR1, DR2, EC1, PE1). The farms were specifically selected to explore the effects of geographical factors and production system. For example, the farms with a conventional production system, but from different countries, could be used to compare the effects of geographical origins. Whereas the farms with different production systems, but from the same country, allow one to determine the effect of production systems. Figure 2 indicates the differences in geographical factors such as altitude, temperature and rainfall due to the different locations. The ten farms occupied different spatial locations on the scatterplot composed of altitude, temperature and rainfall. For example, the farm CR2 from Costa Rica had the highest annual rainfall of 5014 mm/year and an altitude of 47 m. Therefore, it grouped separately from Farm PE1 with a rainfall of 200 mm/year and an altitude of 40 m. However, farms EC2, DR1 and DR2 grouped closely together as they share similar geographical factors.

### 3.2. Differences of Stable Isotopes between Bananas from Different Growing Conditions

For the banana pulp samples, as shown in Table 2, the three stable isotopes ratios (δ^13^C, δ^15^N and δ^18^O) of bananas from different countries in Latin America were compared. For all ten farms, the δ^13^C of pulp samples ranged from −23.8‰ (CR2) to −22.6‰ (PE1). The δ^13^C value for PE1 was significantly different to the carbon isotope ratio of CR3, EC2 and PA1. For the δ^15^N value, the results of DR2 (6.3 ± 1.3‰) was remarkedly higher than the ratios for the other farms. In fact, the δ^15^N values for the conventional farms (CR1, CR3, EC2 and PA1) were significantly lower than most of the organic farms such as DR1, DR2, EC1 and PE1. The stable isotope ratios of oxygen were the highest for CR1 (31.9 ± 0.7‰), while EC2 had the lowest value at 28.4 ± 0.5‰.

The stable isotope ratios of the peel samples were consistent with that of the pulp samples. Table 3 shows that the values of δ^13^C fluctuated from −25.6‰ (DR2) to −23.6‰ (DR1 and PE1). The variation among the δ^15^N values of the samples was greater than that of the δ^13^C values. The nitrogen ratios of the organic farms were significantly higher than the conventional farms; such as 8.0 ± 0.4‰ for DR2 and 0.8 ± 0.6‰ for PA1. For the δ^18^O result, the highest value was reported for DR1 (29.9 ± 0.3‰), while the lowest was 25.2 ± 0.5‰ (EC2). The values from different farms changed according to related growing conditions (*p* < 0.05). With regard to method precision, the standard deviations of repeated pulp samples (*n* = 12) of 10 groups were ranged from 0.2–0.8‰ for δ^13^C, 0.1–1.5‰ for δ^15^N and 0.3–1.1‰ for δ^18^O. In the peel samples, the standard deviations of repeated measurements (*n* = 6) were ranged from 0.2–0.8‰ for δ^13^C, 0.2–0.6‰ for δ^15^N and 0.3–1.3‰ for δ^18^O.

There are several studies reporting that the δ^15^N value could be used to determine if a food product were produced organically [31]. It is well-known that the main type of nitrogen isotope in synthesized fertilizers is isotope ^14^N, while the proportion of the heavier isotope ^15^N is much higher than ^14^N in organic fertilizers [32]. This is because the conventional fertilizers are chemically synthesized by N_2_ in the air under high temperature and high pressure, the content of ^15^N in chemical nitrogen fertilizer is equivalent to that in the atmosphere. However, organic fertilizers are derived from animal matter, animal manure, and vegetable matter, etc., therefore, stable isotope ^15^N is continuously enriched in plant and animals due to bioconcentration effects. Due to the enrichment of ^15^N, the abundance of ^15^N in plants or animals is significantly higher than that in air. Therefore, the abundance of ^15^N in organic fertilizers is significantly higher than that in chemical fertilizers [33]. In line with the literature, the pulp and peel samples from organic farms such as DR1, DR2, EC1 and PE1 had generally higher δ^15^N (^15^N/^14^N) values than the samples from some of the other conventional farms. The highest δ^15^N was 6.3‰ and 8.0‰ in pulp and peel samples of farm DR2, respectively. Most of the conventional farms were reported having δ^15^N values lower than 1.0‰ except for CO1 with a nitrogen isotope ratio of 2.9‰ for pulp and 3.8‰ for the peel samples. This could be due to the fact that some farms choose to apply chemical and organic fertilizers simultaneously to improve soil vitality and agricultural sustainability [34]. Therefore, the application of organic fertilizer could also improve the proportion of the stable isotope ^15^N in the conventionally produced bananas of farm CO1.

### 3.3. Differences of Elemental Compositions between Bananas from Different Growing Conditions

More than 20 types of minerals were detected in the bananas by ICP-MS. In total, 10 elements in pulp and 11 elements in peel samples were reported higher than the limit of detection and hence, only these were selected to compare the effects of geographical factors (altitude, monthly mean temperature and annual rainfall) and production system (organic and conventional cultivation). With regard to method precision, the relative standard deviations of pulp samples among all groups for elements, Fe were less than 10%, Cu, Ni, Sr and Zn were less than 20% and Al, Ba, Mn, Mo, Rb were less than 30%. In the peel samples, slightly higher standard deviations were reported, with Ba, Cu, Fe, Mo, Sr, Zn less than 20%, and Al, Cr, Mn, Ni, Rb were ranged from 20% to 40%. The concentrations of elements for the banana pulp and peel samples obtained from the different countries are listed in Table 2 and Table 3 and Tables S1 and S2 in supplementary materials. For the pulp samples, 10 elements (Al, Ba, Cu, Fe, Mn, Mo, Ni, Rb, Sr and Zn) were detected in the samples. The mean concentration of the elements was significantly different among farms. The concentration of Mn was reported as 21.9 mg/kg for CR1, which was higher (*p* < 0.05) than the concentration for DR1 (5.2 mg/kg). The pulp samples from the organic farms, DR1 and DR2, contained a significantly higher concentration of Zn. For the peel samples, 11 elements (Al, Ba, Cr, Cu, Fe, Mn, Mo, Ni, Rb, Sr and Zn) were obtained and were significantly different according to geographical factors and the production system. The elemental profiles of the banana peel samples were quite different to that of the pulp samples; with the concentration of Al, Fe, Mn, Sr, and Zn being higher in the peel samples. The organic farm, PE1, had a notably lower concentration of Fe when compared to other conventional farms (*p* < 0.05). Most of the farms have significantly different concentrations of elements in the peel samples, but there are still a few farms that did not show any significant differences in some elements (*p* > 0.05). As seen in Table 2 and Table 3, the concentrations of the elements of the pulp and peel samples were significantly different among most of the farms. The results were consistent with the findings of a recent paper about the elemental compositions of bananas [35]. Hardisson et al. detected Fe, Cu, Zn, Mn and B in the banana pulp of *Musa* AAA bananas, the same variety used in the current study [36], which is in line with the current results. As reported in comparative research of eight banana cultivars, the trace elements such as Zn, Fe and Mn were also detected in peel samples [37].

The current study systematically described and compared the trace elements of banana pulp and peel from different growing conditions. The ANOVA results indicates significant differences of elements for most of pulp and peel samples. For example, for the pulp samples, the farm DR2 showed the lowest concentration of Al at 5.4 mg/kg, significantly lower than farm DR1 at 23.5 mg/kg, even though both farms are in the same country. Therefore, it is vital to also consider the geographical (or external) factors as opposed to just the “country border” when studies on the geographical origin of foods are performed. For example, the two geographically distant farms, DR2 (organic) and EC2 (conventional), have similar geographical factors in relation to altitude, temperature and rainfall; the scatter plot (Figure 2) showed that the space location of both farms were almost coincident. Likewise, the concentration of most of the elements such as Ba, Cr, Cu, Fe, Mn, Mo, Ni, Rb and Zn of the peel samples were not significantly different, although these two farms have different production systems. Hence, there are likely other external factors that have a greater influence on the variation in elemental compositions of bananas.

### 3.4. Principal Component Analysis of Bananas between the Different Growing Condition

**Figure 3:**
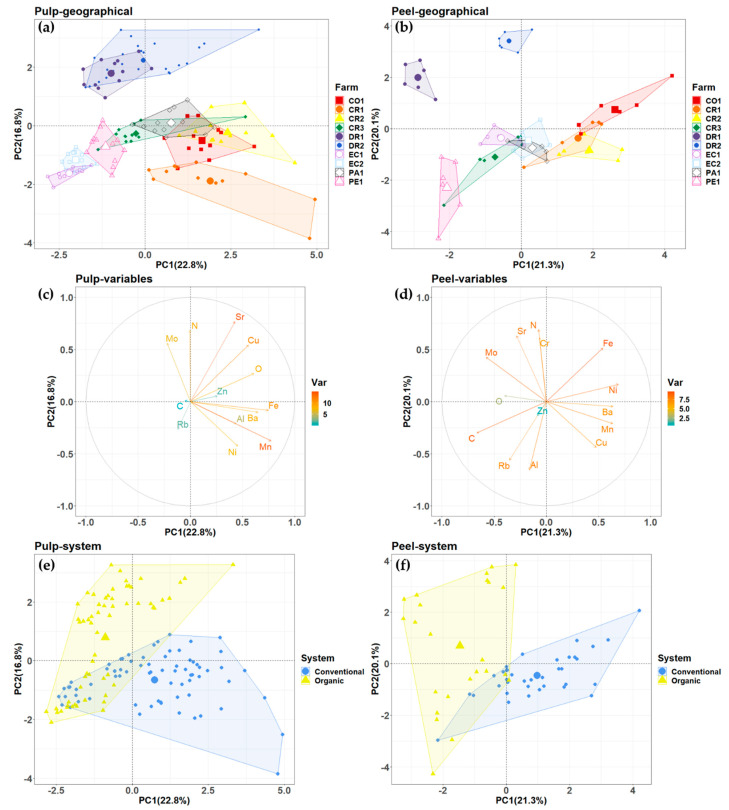
The principal component analysis (PCA) of banana pulp (*n* = 12) and peel (*n* = 6) samples according to stable isotopic ratios and elemental compositions. (**a**) PCA plot showing scores for the pulp samples from the different geographical origins and loadings for the variables. Explained variance: PC1 = 22.8%, PC2 = 16.8%; (**b**) PCA plot showing scores for the pulp samples from the different production systems and loadings for the variables. Explained variance: PC1 = 22.8%, PC2 = 16.8%; (**c**) the loading plot of stable isotopic ratios and elemental compositions for banana pulp samples; (**d**) the loading plot of stable isotopic ratios and elemental compositions for banana peel samples; (**e**) PCA plot showing scores for the peel samples from the different geographical origins and loadings for the variables. Explained variance: PC1 = 21.3%, PC2 = 20.1%; (**f**) PCA plot showing scores for the peel samples from the different production systems and loadings for the variables. Explained variance: PC1 = 21.3%, PC2 = 20.1%.CO: Colombia; CR: Costa Rica; DR: Dominican Republic; EC: Ecuador; PA: Panama; PE: Peru (please refer to the web version of this article for the interpretation of the colors used in the figure).

To explore the effects of growing conditions on bananas compositions, the IR-MS and ICP-MS data sets were subjected to multivariate data analysis. The PCA was used to show the distribution and groupings of the pulp and peel samples (Figure 3). The PCA plot shows that most of the farms could be separated according to differences in the stable isotopic ratios and elemental compositions of the samples. For the pulp and peel samples, PC1 and PC2 explained 39.6% and 41.4%, respectively, of the total variance. Some farms from the same countries, like CR1, CR2, and CR3 from Costa Rica and DR1, DR2 from the Dominican Republic, overlapped in the PCA plots. Figure 3a,b show the farm distribution according to different geographical factors and production system, respectively. DR1 and DR2 were clearly separated from the other farms (Figure 3a). The differences in the concentrations of Mo, N, Sr, Rb and Ni mainly contributed to the farms’ distributions at geographical level. The effects of production system showed that the variables δ^15^N, Mo and Sr were characteristics of organic farms, while Mn, Ni, Al, Ba and Fe were characteristics of conventional farms for the pulp samples (Figure 3c).

The stable isotope ratios and concentration of elements of the peel samples resulted in a different distribution of farms compared to the pulp samples. For instance, the concentration of Sr and Cu show a greater contribution to the discrimination of the peel samples based on geographical origin and production system. As seen in Figure 3e, the peel samples from DR1 and DR2 were grouped separately from samples from Central and South America mainland, with only a slight overlap seen between the different farms. Whereas Figure 3a,c show that the internal variation between peel samples within each farms is lower than their variation in pulp samples, Figure 3c shows that the variables δ^15^N, Mo, Sr and Cr were characteristics of organic farms, while Ni, Ba, Mn, Cu, Al, Rb and Zn were characteristics of conventional farms for the peel samples.

There is considerable evidence that the application of stable isotopes and elements is of great interest for tracing the geographical origin and production system of many food products, such as tea [38], maca [39], orange [40], etc. The use of multi-isotopes (i.e., δ^13^C, δ^15^N) and multi-elements (e.g., Na, Fe, Zn and K) have been reported as reliable tools in authenticity testing of organically grown rice [41]. In the present study, the combination of stable isotopic ratios and elemental compositions were employed for the visualization of multifactorial analysis. The PCA loadings and the contribution of variances are shown in Figure 3c,d. A clear separation was identified between organic and conventional production for the pulp and peel samples. The most important variables driving the separation between groups were δ^13^C, Mo, N, Fe, Ba, Mn and Ni for the pulp samples, and δ^18^O, δ^15^N, Se, Mo, Cu, Al, Mn and Ba for the peel samples. The different contribution of elements in the pulp and peel sample could be because the distribution of minerals in the different parts of the fruits were different. For the results on the geographical origin of the bananas (Figure 3e), the peel samples indicated clearer grouping compared to pulp samples, which could be caused by the lower internal variation between peel samples in the same farm compared with the variation in the pulp samples. The PCA separation was consistent with the results of the ANOVA test of the peel samples, while the above-mentioned elements and stable ratios were also significantly different among the 10 farms (Figure 3d). As seen from Table 2, the farms which showed big differences in the scatter plot (Figure 2), using altitude, temperature and rainfall, also grouped separately in the PCA plots. The relation between the separation in “geographical factors” and “PCA groups” could possibly indicate the effects of growing conditions on the stable isotopic ratios and elemental compositions of bananas. Figure 3 showed that organic farms DR1 and DR2 were separated from other organic farms (EC1 and PE1) in both of pulp and peel results, which could be due to the differences in geographical factors (or other external factors) such as altitude, monthly mean temperature and annual rainfall. For example, the monthly mean temperature of DR1 and DR2 were higher than EC1 and PE1, also the rainfall of DR farms was higher than PE1 and lower than EC1.

At the same time, some values of stable isotopes and elements of organic farms were similar when compared with conventional farms. PCA plots also showed that conventional farms and organic farms were completely separated, such as EC1 and EC2 (Figure 3). This phenomenon could be because organic fertilizers were also used in conventional farms for sustainable banana farming [42], which could explain why some organic farms, such as PE1, were plotted closely to conventional farm such as EC2 in PCA results. Overall, the differences in stable isotope ratios and elemental compositions of bananas from selected countries were the result of the combined effects of the geographical factors and production systems.

### 3.5. Correlation Analysis of Banana Compositions and Their Growing Conditions

To protect the integrity of food and the interests of consumers, most of the related works of literature attempt to discriminate the origin of foods based on the geographical distances such as the “national or provincial boundaries”, whereas only a few papers have been published concerning the underlying mechanisms linked to geographical origin. However, both macromolecular nutrients and micro-metabolites of foods are influenced by the environmental conditions of the origin, planting methods, temperature, soil conditions, etc. [43,44]. Therefore, exploring the correlations between the authentic growing conditions and the composition of foods could providing more in-depth evidence for its traceability. The banana is a suitable fruit to use for correlation research between growing conditions and compositional attributes, as most banana plantations are only located in tropical and subtropical regions, while being sold worldwide. The fresh banana is the most important fruit traded internationally. Verifying the geographical origin of bananas is important to protect the consumers’ pocket and farmers’ benefits [5]. At the same time, identifying and tracing organic bananas (i.e., production system) could further ensure the sustainable supply of bananas and protect consumers’ right to know what they are buying.

**Figure 4:**
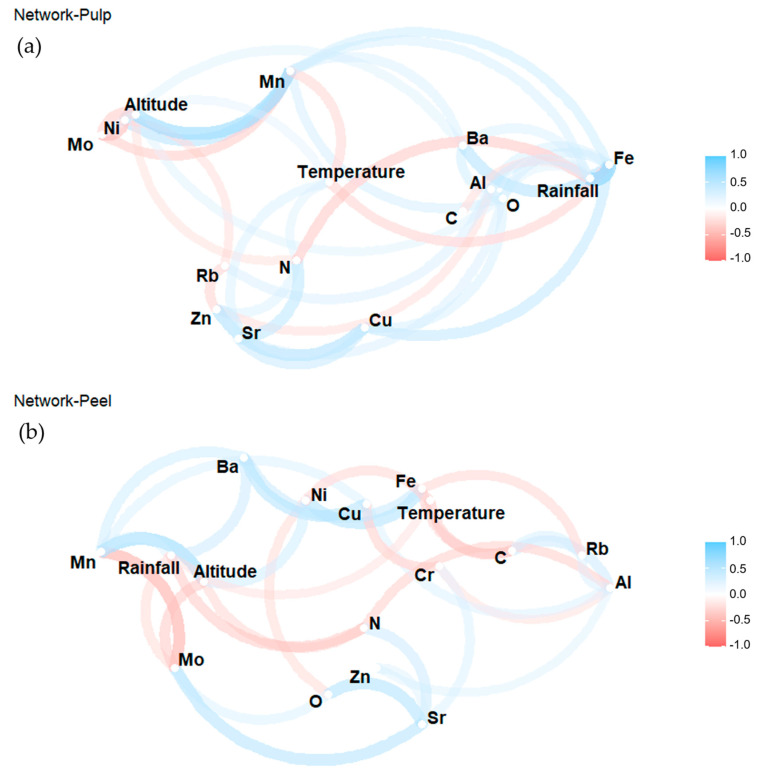
The correlation network of stable isotopes, elements and growing conditions of banana pulp (**a**) and peel (**b**) (refer to the web version of this article for the interpretation of the colors used in the figure).

To further explain the relationships between the stable isotopes and elements of different sampling farms and the local growing conditions, the compositions and their growing conditions were correlated using Pearson’s correlation coefficients (R). The relationship between growing conditions and banana compositions were shown using the network in Figure 4. The distance between the attributes indicated the strength of the correlation coefficient, while the blue or red colors were used to show if they were positive or negative, respectively.

For banana pulp, a slight negative relationship was found in δ^13^C and rainfall (R = −0.32, *p* = 0.18 × 10^−3^), which is in agreement with the several studies where δ^13^C of fruits negatively correlate with the rainfall during sugar accumulation in fruit body [45]. δ^18^O of wine was reported to be highly correlated with daily temperatures and local precipitation [46], and the network analysis of banana pulp showed that δ^18^O was slightly positively correlated with altitude (R = 0.33, *p* = 0. 14 × 10^−3^) and rainfall (R = 0.33, *p =* 0. 12 × 10^−3^) (Figure 4a). Table 1 and Table 2 also showed that farm CR1 had the higher altitude and rainfall values than CO1, at the same time, significant higher δ^18^O were detected in pulp samples from CR1. As for stable isotope N, only a weak positive correlation with temperature and δ^15^N (R = 0.32, *p* < 0.01) were observed. On the other hand, the element Fe indicated a strong correlation with rainfall (R = 0.74, *p* = 0.01), a significant and moderate correlation was found between altitude and two elements: Mn (R = 0.66, *p* = 0.01) and Ni (R = 0.53, *p* < 0.01). A negative correlation with Fe (R = −0.38, *p* < 0.01) and Mn (R = −0.36, *p* < 0.01) due to the effects of temperatures was also found in network analysis. Generally, the differences among sampling locations were mainly caused by individual geographical features such as the altitude, temperature and rainfall. The growth of bananas requires sufficient humidity, light and moisture, otherwise the yield and quality of bananas would be decreased [47].

For banana peel, Figure 4b shows that most of the compositional attributes of peel samples were associated with geographical factors. The differences of δ^13^C from different farms could be associated with temperature conditions (R = −0.41, *p* = 0.90 × 10^−3^), while the variation of δ^15^N was slightly correlated with altitude (R = −0.36, *p* = 0.42 × 10^−2^) and rainfall (R = −0.42, *p* = 0.77 × 10^−3^). It could suggest that other factors likely also contribute to the variations of stable isotope N from different farms. Most of the research indicates that organic production tends to increase the values of δ^15^N more than conventional cultivation [48]. The PCA plots also confirmed that organic farms could have higher values of δ^15^N. The slight negative relationship was found between rainfall and δ^13^C (R = −0.32, *p* = 0.18 × 10^−3^). Similarly, the elements Mn (R = 0.65, *p* < 0.01) and Ni (R = 0.41, *p* = 0.94 × 10^−3^) showed significantly positive correlations with altitude. A slight positive relationship was found in rainfall and Ba (R = 0.44, *p* = 0.04 × 10^−3^).

The significant correlation between growing conditions of the farms and the compositional attributes (stable isotopic ratios and elemental concentrations) was an obvious indication of the relationship between growing conditions and banana compositions. The underlying reason may be that geographical factors (e.g., altitude, temperature, rainfall) affected the absorption capacity of banana roots to elements, thereby promoting or inhibiting the concentration of elements in pulp and peel. Heat stress could induce various physiological injuries to plants such as root growth inhibition, therefore the absorption of elements from soil to roots will be inhibited as well [49]. Subsequently, a negative correlation between temperature and most of the elements of banana pulp and peel were observed in this study. Especially for farm DR1 and DR2 with higher monthly mean temperature than most farms, the elemental contents like Ba, Cu, Fe, Mn, Ni and Cu of pulp samples were also lower than other farms correspondingly. In the effects of altitude on fruit compositions, Crespo et al. reported that chemical composition such as vitamin C, organic acids and anthocyanins of strawberry changed due to increasing altitude [50]. The possible reason could be that the microclimate in a high-altitude region changed a lot in air temperature, precipitation and evaporation compared with a low-altitude region [51]. As found in this study, the altitude also correlated with Mn, Mo, Ni positively or negatively of pulp and peel samples. However, not all stable isotope and elements correlated with the growing conditions, which means more geographical factors such as soil type, sunshine data, etc., should be collected to strengthen the findings.

## 4. Conclusions

A comprehensive application of IR-MS and ICP-MS was employed to identify the isotopes and mineral elements fingerprints of Cavendish bananas from different origins. The ANOVA results demonstrated that the stable isotopic ratios and elemental concentrations changed significantly according to different farm locations. Based on the obtained PCA plots, the selected farms could be separated according to different geographical factors and production systems. This revealed that the variation among banana compositions were potentially related to their growing conditions. Moreover, the δ^15^N values of pulp and peel were significantly higher in the organic system than the conventional system. On the other hand, relations exist between the geographical factors and the concentration of elements in bananas; particularly, Mn and Ni were significantly positively correlated with altitude. The underlying reason could be that the interaction of roots and mineral elements and the banana metabolism is affected by the growing conditions. Overall, the combination of stable isotopes and elemental compositions could be used to explore the effects of local growing conditions concerning origin. However, this feasibility study was limited to one single season and a low number of samples. It is important that more comprehensive discussions on the effects of environmental factors are strengthened in future work. In the following research, a soil map based on correlation of banana composition and growing conditions will be generated to predict the compositional features of bananas without huge chemical database.

## Figures and Tables

**Table 1:** The banana pulp and peel samples collected from different countries and related growing conditions.

Country	Farm	Pulp (*n*)	Peel (*n*)	Altitude (m)	Monthly Mean Temperature (°C)	Annual Rainfall (mm/Year)	Production System
Colombia	CO1	12	6	66	23.2	1837	Conventional
Costa Rica	CR1	12	6	726	23.4	2857	Conventional
	CR2	12	6	47	24.4	5014	Conventional
	CR3	12	6	24	26.3	1837	Conventional
Dominica Republic	DR1	12	6	65	26.7	925	Organic
	DR2	12	6	27	26.7	925	Organic
Ecuador	EC1	12	6	32	22.9	1511	Organic
	EC2	12	6	22	26.5	843	Conventional
Panama	PA1	12	6	20	19.7	3679	Conventional
Peru	PE1	12	6	40	24.1	200	Organic

*n*: number of samples.

**Table 2:** The analysis of variance (ANOVA) of the stable isotopes ratios (^13^C/^12^C, ^15^N/^14^N and ^18^O/^16^O) and elemental compositions (mg/kg) of banana pulp samples (*n* = 12).

Farm	δ^13^C	δ^15^N	δ^18^O	Al	Ba	Cu	Fe	Mn	Mo	Ni	Rb	Sr	Zn
CO1	−23.0 ^ab^ ± 0.8	2.9 ^b^ ± 0.8	30.9 ^bc^ ± 0.5	0.8 ^bcd^ ± 0.2	1.6 ^a^ ± 0.2	3.6 ^cd^ ± 0.5	11.5 ^b^ ± 1.3	15.7 ^b^ ± 3.8	0.1 ^cd^ ± 0.1	0.3 ^a^ ± 0.1	1.9 ^c^ ± 0.3	2.6 ^b^ ± 0.4	6.1 ^bcd^ ± 1.1
CR1	−23.3 ^ac^ ± 0.5	−0.9 ^f^ ± 0.9	31.9 ^a^ ± 0.7	0.9 ^ac^ ± 0.3	0.5 ^b^ ± 0.1	3.9 ^bcd^ ± 0.6	10.7 ^b^ ± 0.9	21.9 ^a^ ± 5.5	0.0 ^d^ ± 0.0	0.3 ^a^ ± 0.1	1.3 ^c^ ± 0.3	2.0 ^bc^ ± 0.5	5.2 ^de^ ± 0.8
CR2	−23.8 ^ac^ ± 0.6	1.1 ^cd^ ± 1.5	31.4 ^ab^ ± 0.6	1.2 ^a^ ± 0.5	1.6 ^a^ ± 0.3	4.5 ^ab^ ± 0.4	12.7 ^a^ ± 0.5	12.9 ^bc^ ± 1.9	0.2 ^b^ ± 0.0	0.1 ^cd^ ± 0.0	9.0 ^b^ ± 1.1	2.5 ^b^ ± 0.6	5.9 ^bce^ ± 1.2
CR3	−23.5 ^bc^ ± 0.4	−0.5 ^ef^ ± 0.6	31.2 ^ac^ ± 0.4	0.9 ^bc^ ± 0.1	0.4 ^bc^ ± 0.0	3.7 ^cd^ ± 0.2	10.7 ^b^ ± 0.6	9.2 ^ce^ ± 2.9	0.2 ^b^ ± 0.1	0.2 ^cd^ ± 0.0	7.0 ^b^ ± 2.0	1.9 ^bc^ ± 0.2	5.9 ^bce^ ± 0.5
DR1	−23.3 ^ac^ ± 0.5	1.9 ^cd^ ± 0.4	30.5 ^c^ ± 0.3	0.6 ^cd^ ± 0.2	0.3 ^cd^ ± 0.0	4.2 ^ac^ ± 0.7	8.8 ^c^ ± 0.7	5.2 ^f^ ± 1.7	0.3 ^a^ ± 0.1	0.1 ^cd^ ± 0.0	1.9 ^c^ ± 0.8	3.3 ^a^ ± 0.6	7.0 ^ab^ ± 0.6
DR2	−23.2 ^ac^ ± 0.6	6.3 ^a^ ± 1.3	30.9 ^bc^ ± 1.1	0.6 ^cd^ ± 0.3	0.5 ^bc^ ± 0.2	4.5 ^bd^ ± 0.8	9.3 ^c^ ± 0.9	7.6 ^ef^ ± 2.5	0.2 ^b^ ± 0.1	0.1 ^d^ ± 0.0	2.9 ^c^ ± 1.8	3.5 ^a^ ± 0.6	7.1 ^a^ ± 1.1
EC1	−23.1 ^ac^ ± 0.6	1.5 ^ce^ ± 0.6	29.1 ^d^ ± 0.7	0.7 ^cd^ ± 0.2	0.2 ^d^ ± 0.1	2.8 ^e^ ± 0.2	8.6 ^c^ ± 0.5	5.6 ^ef^ ± 0.8	0.1 ^d^ ± 0.0	0.2 ^bc^ ± 0.0	7.3 ^b^ ± 2.6	1.2 ^de^ ± 0.4	6.0 ^bce^ ± 0.4
EC2	−23.6 ^bc^ ± 0.2	−0.4^ef^ ± 0.2	28.4 ^d^ ± 0.5	0.4 ^d^ ± 0.1	0.3 ^bd^ ± 0.0	3.6 ^cd^ ± 0.4	8.8 ^c^ ± 0.5	5.3 ^ef^ ± 1.2	0.2 ^bc^ ± 0.0	0.2 ^b^ ± 0.0	1.8 ^c^ ± 0.4	1.0 ^e^ ± 0.1	6.0 ^bcd^ ± 0.3
PA1	−23.4 ^bc^ ± 0.2	0.0 ^def^ ± 0.1	31.0 ^ac^ ± 0.4	0.8 ^bc^ ± 0.2	0.5 ^bd^ ± 0.1	4.7 ^a^ ± 0.4	12.7 ^a^ ± 0.8	11.6 ^cd^ ± 4.2	0.2 ^b^ ± 0.0	0.1 ^d^ ± 0.0	3.4 ^c^ ± 1.5	2.0 ^bc^ ± 0.4	6.8 ^ac^ ± 0.5
PE1	−22.6 ^a^ ± 0.3	1.7 ^c^ ± 0.3	31.5 ^ab^ ± 0.8	1.1 ^ab^ ± 0.2	0.4 ^bc^ ± 0.1	3.4 ^de^ ± 0.2	8.7 ^c^ ± 0.7	8.0 ^def^ ± 2.0	0.2 ^bc^ ± 0.0	0.1 ^d^ ± 0.0	12.1 ^a^ ± 5.2	1.7 ^cd^ ± 0.2	5.0 ^e^ ± 0.3

^a–f^ Different mean values in the same column with different superscript letters are significantly different (*p* < 0.05) according to Tukey’s significant difference test; the Cr was not presented because of values lower than limit of detection.

**Table 3:** The analysis of variance (ANOVA) of stable isotopes ratios (^13^C/^12^C, ^15^N/^14^N and ^18^O/^16^O) and elemental compositions (mg/kg) of banana peel samples (*n* = 6).

Farm	δ^13^C	δ^15^N	δ^18^O	Al	Ba	Cr	Cu	Fe	Mn	Mo	Ni	Rb	Sr	Zn
CO1	−25.0 ^bd^ ± 0.8	3.8 ^b^ ± 0.3	25.7 ^cd^ ± 0.3	24.3 ^bc^ ± 2.6	8.7 ^b^ ± 1.1	0.1 ^ab^ ± 0.0	5.1 ^ab^ ± 0.6	24.8 ^a^ ± 9.3	43.2 ^bcd^ ± 7.8	0.2 ^bc^ ± 0.1	0.4 ^a^ ± 0.1	3.1 ^d^ ± 0.7	22.0 ^c^ ± 3.5	17.8 ^ab^ ± 1.7
CR1	−24.7 ^bd^ ± 0.5	−1.1 ^f^ ± 0.5	28.0 ^b^ ± 1.0	23.5 ^cd^ ± 7.5	3.6 ^cd^ ± 0.6	0.1 ^ab^ ± 0.1	4.0 ^cd^ ± 0.3	19.6 ^ac^ ± 1.4	82.2 ^a^ ± 15.6	0.1 ^d^ ± 0.0	0.3 ^ab^ ± 0.1	6.0 ^d^ ± 4.7	20.8 ^cd^ ± 3.1	17.8 ^ab^ ± 2.0
CR2	−25.0 ^bd^ ± 0.3	0.5 ^df^ ± 0.3	27.9 ^b^ ± 1.3	24.7 ^bc^ ± 5.9	10.6 ^a^ ± 1.5	0.1 ^ab^ ± 0.0	5.8 ^a^ ± 0.7	21.8 ^ab^ ± 1.2	61.7 ^ab^ ± 26.9	0.2 ^bc^ ± 0.0	0.2 ^b^ ± 0.0	24.3 ^bc^ ± 7.2	22.3 ^c^ ± 8.5	15.7 ^ab^ ± 1.9
CR3	−24.6 ^bc^ ± 0.3	−0.3 ^ef^ ± 0.2	28.2 ^b^ ± 0.9	29.1 ^bc^ ± 13.6	3.4 ^cd^ ± 1.4	0.0 ^b^ ± 0.0	4.5 ^bc^ ± 0.8	16.2 ^bcd^ ± 4.7	35.0 ^ce^ ± 11.3	0.2 ^bc^ ± 0.0	0.2 ^c^ ± 0.1	16.4 ^bd^ ± 3.9	21.8 ^c^ ± 4.4	16.5 ^ab^ ± 1.6
DR1	−23.6 ^a^ ± 0.5	2.6 ^c^ ± 0.4	29.8 ^a^ ± 0.3	23.5 ^cd^ ± 4.8	2.4 ^cd^ ± 0.3	0.1 ^ab^ ± 0.0	3.7 ^cd^ ± 0.6	19.9 ^ac^ ± 2.5	18.0 ^ce^ ± 6.9	0.5 ^a^ ± 0.1	0.2 ^c^ ± 0.0	8.8 ^cd^ ± 5.3	41.5 ^a^ ± 4.6	19.0 ^a^ ± 2.5
DR2	−25.6 ^d^ ± 0.5	8.0 ^a^ ± 0.4	27.2 ^bc^ ± 1.3	5.4 ^e^ ± 1.4	3.0 ^cd^ ± 0.1	0.1 ^a^ ± 0.0	3.4 ^d^ ± 0.4	20.5 ^ac^ ± 2.4	31.7 ^ce^ ± 2.5	0.3 ^b^ ± 0.0	0.2 ^c^ ± 0.0	11.8 ^cd^ ± 5.3	33.7 ^b^ ± 1.9	15.3 ^ab^ ± 2.7
EC1	−24.4 ^ab^ ± 0.5	1.5 ^cde^ ± 0.5	26.7 ^bd^ ± 0.7	11.1 ^de^ ± 2.3	1.8 ^d^ ± 0.4	0.1 ^ab^ ± 0.0	3.6 ^cd^ ± 0.4	16.5 ^bcd^ ± 1.0	28.0 ^ce^ ± 3.6	0.1 ^cd^ ± 0.0	0.3 ^bc^ ± 0.0	28.7 ^b^ ± 5.2	13.3 ^de^ ± 2.4	15.5 ^ab^ ± 1.4
EC2	−24.9 ^bd^ ± 0.3	0.5 ^df^ ± 0.2	25.2 ^d^ ± 0.5	21.3 ^cd^ ± 4.3	2.1 ^cd^ ± 0.1	0.1 ^ab^ ± 0.0	4.2 ^bd^ ± 0.3	16.3 ^bcd^ ± 1.4	21.2 ^de^ ± 7.0	0.2 ^bc^ ± 0.0	0.3 ^bc^ ± 0.1	10.8 ^cd^ ± 7.6	10.5 ^e^ ± 2.1	14.3 ^b^ ± 1.2
PA1	−25.3 ^cd^ ± 0.3	0.8 ^cde^ ± 0.6	28.4 ^ab^ ± 0.8	19.8 ^cd^ ± 1.7	2.6 ^cd^ ± 0.6	0.0 ^b^ ± 0.0	5.6 ^a^ ± 0.5	13.7 ^cd^ ± 0.8	45.8 ^bc^ ± 18.7	0.2 ^bc^ ± 0.0	n.d.	11.9 ^cd^ ± 6.0	23.5 ^c^ ± 3.8	19.0 ^a^ ± 1.9
PE1	−23.6 ^a^ ± 0.2	1.9 ^cd^ ± 0.5	27.4 ^b^ ± 0.8	37.3 ^b^ ± 11.7	2.9 ^cd^ ± 0.2	0.1 ^ab^ ± 0.0	4.4 ^bc^ ± 0.5	12.2 ^d^ ± 1.2	34.0 ^ce^ ± 4.5	0.2 ^b^ ± 0.0	0.2 ^c^ ± 0.1	47.7 ^a^ ± 20.5	17.0 ^ce^ ± 1.8	18.2 ^a^ ± 1.9

^a–f^ Different mean values in the same column with different superscript letters are significantly different (*p* < 0.05) according to Tukey’s significant difference test; n.d.: none detected.

## Data Availability

Not available.

## Supplementary Materials

The following are available online at https://www.mdpi.com/article/10.3390/foods10051021/s1, Table S1: The minimum and maximum values of the stable isotopes ratios (^13^C/^12^C, ^15^N/^14^N and ^18^O/^16^O) and elemental compositions (mg/kg) of banana pulp samples (*n* = 12). Table S2: The minimum and maximum values of the stable isotopes ratios (^13^C/^12^C, ^15^N/^14^N and ^18^O/^16^O) and elemental compositions (mg/kg) of banana pulp samples (*n* = 6).
